# Kinetics of Hypotension during 50 Sessions of Resistance and Aerobic Training in Hypertensive Patients: a Randomized Clinical Trial

**DOI:** 10.5935/abc.20170029

**Published:** 2017-04

**Authors:** Igor Rodrigues Damorim, Tony Meireles Santos, Gustavo Willames Pimentel Barros, Paulo Roberto Cavalcanti Carvalho

**Affiliations:** Programa de Pós-Graduação em Educação da Física da Universidade Federal de Pernambuco - UFPE, Recife, PE - Brazil

**Keywords:** Hypertension, Kinetics, Exercise, Exercise Movement Techniques, Clinical Trial

## Abstract

**Background:**

Resistance and aerobic training are recommended as an adjunctive treatment for hypertension. However, the number of sessions required until the hypotensive effect of the exercise has stabilized has not been clearly established.

**Objective:**

To establish the adaptive kinetics of the blood pressure (BP) responses as a function of time and type of training in hypertensive patients.

**Methods:**

We recruited 69 patients with a mean age of 63.4 ± 2.1 years, randomized into one group of resistance training (n = 32) and another of aerobic training (n = 32). Anthropometric measurements were obtained, and one repetition maximum (1RM) testing was performed. BP was measured before each training session with a digital BP arm monitor. The 50 training sessions were categorized into quintiles. To compare the effect of BP reduction with both training methods, we used two-way analysis of covariance (ANCOVA) adjusted for the BP values obtained before the interventions. The differences between the moments were established by one-way analysis of variance (ANOVA).

**Results:**

The reductions in systolic (SBP) and diastolic BP (DBP) were 6.9 mmHg and 5.3 mmHg, respectively, with resistance training and 16.5 mmHg and 11.6 mmHg, respectively, with aerobic training. The kinetics of the hypotensive response of the SBP showed significant reductions until the 20th session in both groups. Stabilization of the DBP occurred in the 20th session of resistance training and in the 10th session of aerobic training.

**Conclusion:**

A total of 20 sessions of resistance or aerobic training are required to achieve the maximum benefits of BP reduction. The methods investigated yielded distinct adaptive kinetic patterns along the 50 sessions.

## Introduction

The practice of physical exercise is the most used strategy for nonpharmacological treatment of hypertension.^1,2^ Aerobic stimuli between 40-60% of the maximum oxygen consumption (VO_2max_) are recommended two to three times a week during sessions of 30 to 60 minutes, performed in association with resistance training using multiarticular exercises with at least one series of 8-12 repetitions for 30 to 60 minutes.^3^

Reductions of 6.9 mmHg in the systolic BP (SBP) and 4.9 mmHg in the diastolic BP (DBP) during rest have been reported as a result of adaptations enabled by aerobic training.^4^ Although aerobic training is the most established strategy among the methods of physical training for hypertensive individuals, other methods have been shown to be effective in reducing BP levels, such as resistance dynamic,^5^ isometric,^6^ combined (aerobic and resistance),^7^ and high-intensity interval training.^8^

 Studies with resistance training as the only nonpharmacological strategy to treat hypertension have demonstrated BP reductions between 2 and 12 mmHg.^9,10^ Even after interruption, the effects of training persist for up to 4 weeks.^11^

To the best of our knowledge, available studies directly comparing different training methods, such as aerobic *versus* resistance training,^12,13^ have not identified the number of sessions required until stabilization of the hypotensive effect of the exercise in hypertensive patients. More precisely, it is important to clarify how many sessions are necessary to ensure that the training programs provide the maximum possible benefits. This outcome has not been investigated with priority, and the results regarding the number of sessions are still inconclusive in the literature (between 12 to 48 sessions),^14^ hindering the interpretation of the adjustments provided by different methods of training and the consequent decision for the best treatment strategy.^15^

Thus, the objective of this study was to establish the adaptive kinetics of the BP responses as a function of time and type of training (resistance or aerobic) in individuals classified with stage 1 hypertension.

## METHOD

### Experimental design

Clinical trial with two parallel groups conducted according to the CONSORT recommendations, but without registration. Eligible subjects were randomized into two independent training groups: resistance and aerobic. On the first visit, the subjects received instructions regarding the procedures of the study, had their questions answered, and signed a free and informed consent form (ICF). On the second visit, anthropometric and BP measurements were obtained. On the third visit, one repetition maximum (1RM) testing was performed in the resistance group, and recommendations regarding the prescription of training were delivered in the aerobic group. On the fourth visit, adaptations of the participants to their respective training methods were made. From the fifth visit onwards, the training protocols were carried out in both groups.

### Subject

We recruited for the study 20 men and 49 women, whose characteristics are described in Table 1. All subjects participated voluntarily after being contacted through invitations and reports on the practice of physical activity for hypertensive patients, distributed on the campus of the *Universidade Federal de Pernambuco*. All participants used medication for BP control (Table 2). The research was approved by the Ethics Committee at *Centro de Ciências da Saúde* at *Universidade Federal de Pernambuco* (case 321/11).

**Table 1:** General characteristics of the investigated subjects before training

Variables	Resistance Group	Aerobic Group
Age (years)	62.8 ± 1.22	63.9 ± 2.3
Weight (kg)	69.2 ± 13.7	70.6 ± 11.5
SBP	147.0 ± 9.4	151.8 ± 11.5
DBP	95.8 ± 7.9	93.9 ± 10.8
BMI (kg.m^-2^)	30.3 ± 30.1	29.2 ± 4.7
WHR	0.95 ± 0.21	0.90 ± 0.76
CI	1.55 ± 0.11	1.56 ± 0.23
WC (cm)	98.2 ± 6.0	97.9 ± 13.1
AC (cm)	102.0 ± 9.4	99.2 ± 12.3

SBP: systolic blood pressure; DBP: diastolic blood pressure; BMI: body mass index; WHR: waist-hip ratio; CI: conicity index; WC: waist circumference; AC: abdomen circumference.

**Table 2:** Frequency and percentage of medications used by the participants

Antihypertensive drugs	Resistance Group	Aerobic Group	Total Frequency
	(n = 28)	(n = 27)	(n = 55)
Angiotensin converting enzyme inhibitors	5 (55%)	4 (45%)	9 (16%)
Diuretics	5 (45%)	6 (55%)	11 (20%)
Angiotensin receptor II antagonists	15 (50%)	15 (50%)	30 (55%)
Calcium channel antagonists	3 (60%)	2 (40%)	5 (9%)

As the inclusion criteria, the subjects should have stage 1 hypertension, use controlled medications, and be older than 60 years. On the first visit, we measured the participants' BP at rest, which was considered as the initial reference (moment 0) and was used to classify the subjects regarding their hypertension level.^16^

**Figure 1:**
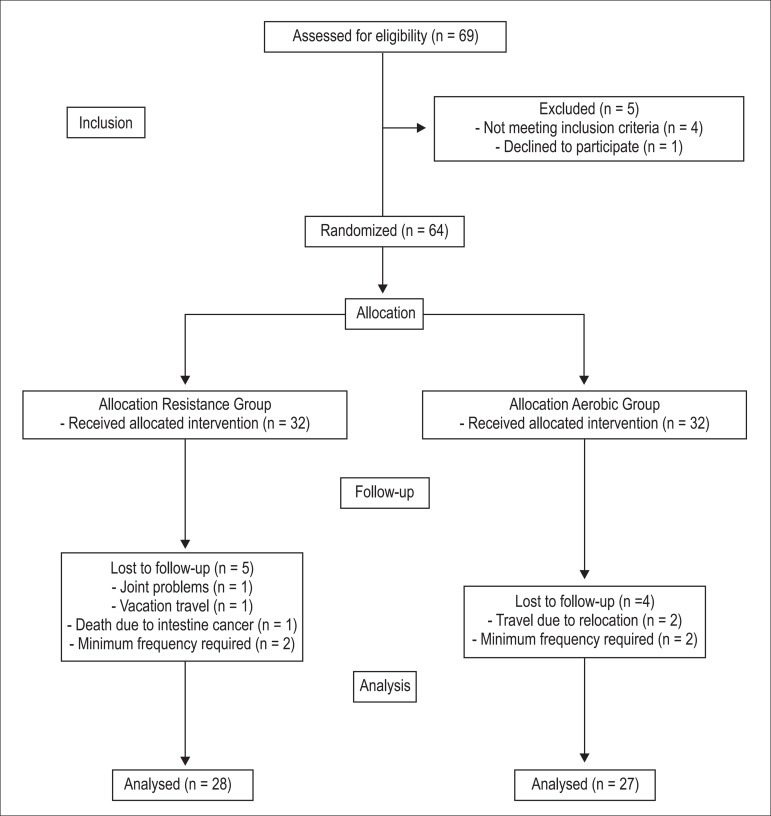
Flow diagram of the randomization of the subjects.

We excluded subjects using beta-blockers, since this type of medication changes the individual's cardiovascular responses, hindering the interpretation of the data and the use of the heart rate to prescribe training.^17^ We also excluded participants who had any other disease affecting cardiovascular responses to physical exercise, or with joint limitations resulting in functional limitations. Figure 1 shows the flowchart of the subjects throughout the study.

For randomization, we used a digital tool available at www.randomizer.org. The eligible subjects were listed numerically in order of arrival by one of the researchers without access to any of the evaluations. A second researcher was blindly responsible for the allocation of the participants to each group.

### Procedures

#### Anthropometric assessments and weight indices

We measured the participants' body mass (kg), height (cm), and waist and hip circunferences (cm). Body mass was measured using a portable scale accurate to 0.1 kg (PL 200, Filizola S.A., São Paulo, Brazil). The height was measured with a stadiometer accurate to 0.1 cm (Professional Stadiometer Sanny, São Paulo, Brazil). The waist circumference was measured at the narrowest level between the rib margin and the iliac crest using a non-flexible anthropometric tape precise to 0.1 mm (SN-4010, Sanny, São Paulo, Brazil). The hip circumference was measured at the level of the pubic symphysis using the same tape. We then calculated the subjects' body mass index (BMI = body weight ÷ height^2^), their waist/hip ratio (WHR = waist circumference ÷ hip circumference), and their conicity index [CI = (circumference of the abdomen ÷ 0.169) x √(body weight ÷ height)].^18^

#### Blood pressure measurement

The BP was measured at rest in the left superior limb according to recommendation by the American Heart Association, using a digital BP monitor (Digital Omron BP Monitor, Model 11 EM403c, Tokyo, Japan). Considered as the primary outcome in the present study, the BP was monitored before each training session, and the last measurement was performed 48 h after the 50th session. The subjects were instructed to not drink alcohol and/or caffeine for 24 h before the measurements. For each measurement, the subjects rested for 15 min in the sitting position with their feet supported and kept their arm at the heart level.

#### One repetition maximum testing

The 1RM test was performed according to the protocol of the American College of Sports Medicine.^3^ For that, the subjects performed warm-up exercises with 10 repetitions with a light load. After 5 min, the 1RM load testing was carried out, in which each subject performed at the most five attempts of each exercise with an interval of 5 min between each one, in which the largest lifted load was the load selected.

#### Resistance training protocol

The resistance training sessions were carried out on exercise equipment (Technogym, Cesena, Italy). The subjects performed a program of resistance training alternated by segment, with two types of series (A and B), alternated by session (48 h). The order of the exercises was: A series - vertical bench press, seated leg curl, triceps cable curl, seated leg abduction, shoulder lift, plantar flexion, and upper abdominal; B series - frontal cable pull, leg press, shoulder abduction, leg extension, biceps curl, seated leg adduction, and lower abdominal. The training program was performed three times a week, with three sets of 12 repetitions at 50-70% of the maximum load and adjusted throughout the program for the achievement of a perceived exertion (Borg) classified as moderate. A 1 min recovery between each series and exercises was administered.

#### Aerobic training protocol

The sessions of aerobic training consisted of walking on track three times a week for 30 min, maintaining the heart rate between 40-60% of the predicted maximum rate for age.^19^ The intensity was adjusted over the course of the sessions based on the participant's subjective perception of effort, aiming to reach a moderate intensity. All training sessions were supervised.

### Statistical analysis

Quantitative variables are presented as mean ± standard deviation. Categorical variables are presented by their absolute and relative frequencies. The 50 training sessions were divided into quintiles, yielding five comparative moments (sessions 1-10, 11-20, 21-30, 31-40, 41-50). The BP result at each quintile represents the average of 10 sessions grouped for each variable investigated (SBP and DBP) measured before each training session. The pretreatment measurement of the dependent variables was used as a covariate to control the initial differences between the groups. Given the possibility of sampling mortality, the analyses conducted were not based on an "intention to treat". After verifying the conceptual assumptions, to compare the effect of the methods of resistance and aerobic training on the SBP and DBP measurements, we used two-way analysis of covariance (ANCOVA; training method x moment) with repeated measures for the second factor.

The identification of the differences between the investigated moments for each training method was established with one-way analysis of variance (ANOVA) with repeated measures. For both analyses, we used the *post hoc* Bonferroni test, when necessary. The analyses were performed using GraphPad Prism, v. 5.0 (GraphPad Software, San Diego, USA), with a significance level set at p < 0.05.

## Results

**Figure 2:**
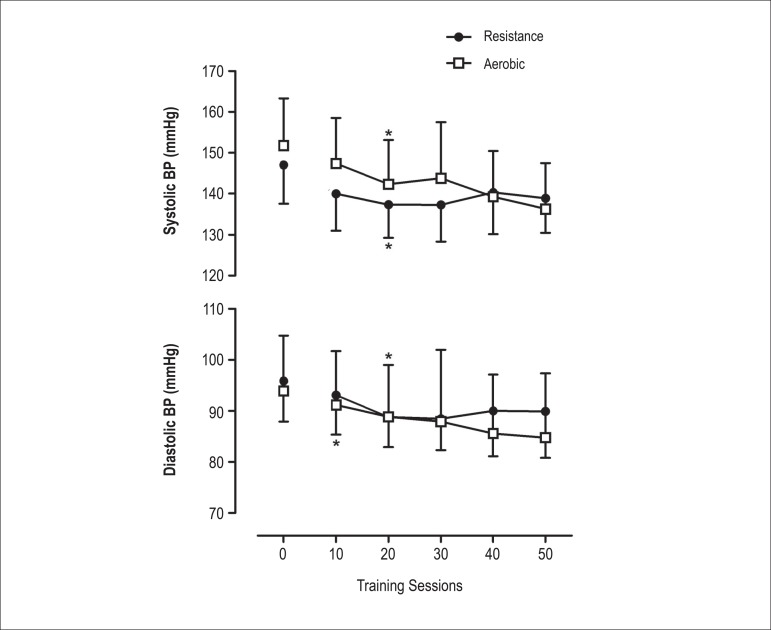
Responses in systolic and diastolic blood pressure at rest obtained before the exercise sessions in the resistance and aerobic groups. BP: Blood Pressure.

We performed preliminary verifications to ensure that there was no violation of the assumptions of normality, linearity, variance homogeneity, regression slope homogeneity, and reliable covariate measurement. Figure 2 shows a comparison of the BP along the 50 sessions of resistance and aerobic training, and Table 3 highlights the differences (Δ) observed and their respective confidence intervals. ANCOVA indicated a significant interaction between the training methods in regards to the SBP (F [4, 29] = 3.431, p = 0.021), with a small *eta squared* effect size (η^2^ = 0.321). The analysis of the main effects showed no significant differences between the training methods in terms of SBP (p = 0.690); however, the results suggested that the SBP responded with different reductions in both groups.

**Table 3:** Difference (Δ), standard deviation, and confidence intervals of the hypotensive responses of the systolic blood pressure (SBP) and diastolic blood pressure (DBP) at five different moments in the resistance and aerobic groups

Blood Pressure	Resistance Group			Aerobic Group	
	Mean ± SD	95%CI		Mean ± SD	95%CI
**Systolic**					
Δ 10-0	-7 ± 0.4	-7.2; -6.8		-4.4 ± 0.34	-4.6; -4.2
Δ 20-0	-9.7 ± 8.7	-14.0; -5.4		-9.5 ± 6.1	-13.0; -6.4
Δ 30-0	-9.7 ± 6.1	-13.0; -6.7		-8.0 ± 9.2	-13.0; -3.3
Δ 40-0	-6.7 ± 7.2	-10.0; -3.1		-13.0 ± 9.2	-17.0; -7.8
Δ 50-0	-8.2 ± 8.4	-12.0; -4.0		-16.0 ± 9.2	-20.0; -11.0
**Diastolic**					
Δ 10-0	-2.8 ± 0.2	-2.9; -2.7		-2.7 ± 0.3	-2.9; -2.6
Δ 20-0	-7.1 ± 5.6	-9.9; -4.3		-5.1 ± 7.0	-8.7; -1.5
Δ 30-0	-7.4 ± 6.1	-10.0; -4.4		-6.0 ± 9.2	-11.0; -1.3
Δ 40-0	-5.9 ± 8.4	-10.0; -1.7		-8.3 ± 7.7	-12.0; -4.4
Δ 50-0	-6.0 ± 8.0	-10.0; -2.0		-9.2 ± 8.6	-14.0; -4.7

Δ - Difference between the moments 10, 20, 30, 40, and 50 in regard to moment 0. CI: Confidence interval; SD: Standard deviation.

The interaction between the training methods in regards to the DBP showed an absence of statistically significant results (F [4, 29] = 1.835, p = 0.149), with a small effect size (η^2^ = 0.202). In the analysis of the main effects in the DBP (p = 0.091), the groups responded in a similar manner.

The identification of the moments of BP stabilization as a result of the training strategies is presented in Table 4 for the SBP and in Table 5 for the DBP. The stabilization of the reductions in the SBP was observed in the 20th session for both methods. For the DBP, the reductions were significant until the 20th session of resistance training and up to the 10th session of aerobic training.

**Table 4:** Indicator matrix of statistical significance of one-way analysis of variance (ANOVA) (within) with post hoc Bonferroni for systolic blood pressure comparisons at different moments

Moment	Resistance Group						Aerobic Group				
	10	20	30	40	50		10	20	30	40	50
0	NS	< 0.001	< 0.001	NS	< 0.01		NS	< 0.001	< 0.01	< 0.001	< 0.001
10	---	NS	NS	NS	NS		---	NS	NS	< 0.05	< 0.001
20	---	---	NS	NS	NS		---	---	NS	NS	NS
30	---	---	---	NS	NS		---	---	---	NS	NS
40	---	---	---	---	NS		---	---	---	---	NS

**Table 5:** Indicator matrix of statistical significance of one-way analysis of variance (ANOVA) (within) with post hoc Bonferroni for diastolic blood pressure (DBP) comparison at different moments

Moment	Resistance Group						Aerobic Group				
	10	20	30	40	50		10	20	30	40	50
0	NS	< 0.01	< 0.001	< 0.05	< 0.05		< 0.05	< 0.001	< 0.001	< 0.001	< 0.001
10	---	NS	NS	NS	NS		---	NS	NS	NS	NS
20	---	---	NS	NS	NS		---	---	NS	NS	NS
30	---	---	---	NS	NS		---	---	---	NS	NS
40	---	---	---	---	NS		---	---	---	---	NS

## Discussion

The present study demonstrated that resistance training was able to reduce the SBP in 6.9 ± 2.8 mmHg and the DBP in 5.3 ± 1.9 mm Hg, while aerobic training showed reductions of 16.5 ± 3.4 mmHg in SBP and 11.6 ± 3.6 mmHg in DBP. The interaction between the methods investigated indicates apparently higher hypotensive effects with aerobic training when compared with resistance training. However, the comparison of the mean standardized reductions between the methods by the analysis of the η^2^ showed a small magnitude for both strategies. In the temporal analysis of the training methods, we observed that the kinetics of the hypotensive response of the SBP showed significant reductions until the 20th session in both groups. After that, there was a plateau in the adaptations yielded by resistance training. This is a novel information that should be considered in therapeutic decisions using exercise as an adjuvant in BP treatment.

Even though a statistically significant difference occurred after the 40th session, a regression of the SBP to mean values close to those of the 10th session seems to have occurred. The mechanisms underlying such adaptation could not be identified. Future studies should investigate the hypothesis of the increased arterial stiffness generated by resistance training, as suggested by Okamoto et al.^20^ In addition, aerobic training maintained nonsignificant reductions until the 50th session, which may clinically represent some treatment benefit, especially in patients within the classification limit of a given category (borderline), since an SBP reduction of 10 mmHg reduces the mortality risk by 13%.^21^

In a similar way, we observed that resistance training yielded a significant DBP reduction until the 20th session, while with aerobic training the stabilization occurred after the 10th session. Together, these results provide a better understanding of the adaptive behavior of the SBP and DBP as a result of the investigated training methods, since they provided different kinetic responses.

The physiological mechanisms explaining the BP reductions after physical exercise are, on the one hand, due to a decrease in cardiac output following a reduction in the systolic volume and heart rate and a decrease in the sympathetic tone^22^ and, on the other hand, due to an increase in the baroreflex sensitivity and control, associated with a peripheral local action, mediated mainly by nitric oxide released in the endothelium as a result of stress generated by physical exercise (shear stress).^23^ Together, these mechanisms trigger adaptations such as arterial vasodilation, generating a reduction in peripheral resistance and, consequently, in BP after physical exercise.^24^ For example, Santana et al.^25^ subjected hypertensive elderly women to aerobic exercise with one session at moderate intensity for 20 min and another session at high intensity for 20 min. Nitric oxide levels after the activity increased by 30% and 33%, respectively, and there was a significant reduction in BP with both interventions.

In a recent meta-analysis that investigated the effect of different exercise methods on the magnitude of the effect in reducing the BP, Cornelissen and Smart^26^ did not find differences in effect size between aerobic and resistance training, concluding that both training methods provide BP reductions of similar magnitude. Furthermore, the results reported by the authors presented larger reductions with aerobic training. Both aspects were similar to those found in the present study. In addition, the results of the present study add information to these findings, setting the kinetic standard of BP responses yielded by the two investigated training methods. Future studies should investigate other training strategies.

About the kinetics of BP stabilization, we identified only one study using resistance training,^27^ in which the SBP stabilized at the 6th training session, while in our study we observed significant reductions until the 20th training session. For the DBP, the same study found that the stabilization occurred in the 30th session, while in our study it occurred in the 20th session. It is possible that the differences encountered are the result of the difference in data sampling, since the present study considered the training sessions grouped into quintiles. It is noteworthy that the protocols of resistance training in both studies were similar and were performed with moderate loads (between 50-70% of the 1RM load), with three sets of 12 repetitions.

Regarding aerobic training, Kokkinos et al.^28^ compared the BP responses after 48 and 96 training sessions to the initial BP values, observing a nonsignificant decrease of 1.0 ± 4.0 mmHg (p = 0.150), but with a substantial reduction in the use of medications. On the other hand, Seals and Reiling^29^ found BP reductions in elderly individuals after 72 sessions of aerobic training. Later, when 72 additional sessions of aerobic training were performed, there was an additional SBP reduction of 4.0 ± 4.0 mmHg (p < 0.05), but no DBP reductions. Jennings et al.^30^ found a BP decrease at the 30th session of aerobic training, which corresponded to 75% of the hypotensive effect at the 60th session. This same proportion was found in the present study. Together, this evidence shows that the results of physical exercises on BP treatment in the long term seem to bring benefits only in the maintenance of the initial reductions and do not result in additional gains.

Although resistance training generates smaller reductions when compared with aerobic training,^26^ its recommendation is supported by the reduction in BP responses in daily life activities, since the improvement in resistance promotes a relative reduction in the intensity in which daily tasks are performed, with consequent mitigation of BP responses. Considering that, resistance training seems to be a relevant strategy for BP control and maintenance of functional aspects. One should also consider that, in the light of the available knowledge, the clinical effects of BP reduction by resistance training are similar to those observed with aerobic training.

Some limitations of the present study need to be highlighted. The study did not take into account the doses of the medications used by each subject, which may have influenced the responses observed. However, this approach presents greater external validity considering that the individuals exercising in centers of physical activity and exercise clinics do not interrupt the use of their medications to practice their physical activities. In addition, physical exercise is considered an adjuvant treatment and should be performed along with the use of medications, which should be frequently evaluated for possible adjustments. Another limitation was the lack of use of ambulatory BP monitoring, which enables a more reliable measurement by evaluating the BP levels for a longer period of time. And finally, the absence of a control group limits the conclusion that it was only the exercise that determined the BP decrease. However, prior evidence has established with certainty the benefits of an exercise group (aerobic and resistance) in relation to a control group,(^24,28^) which would characterize as ethically questionable the decision to deprive a group of individuals from exercise treatment.

## Conclusions

We observed that 20 sessions of resistance or aerobic training are necessary to achieve BP reductions resulting from physical exercise, and that the BP reductions respond differently over the course of 50 sessions. A mean reduction per session of 0.5 mmHg in the SBP for both training methods, and 0.2 to 0.3 mmHg in the DBP for resistance and aerobic training, respectively, can be expected up to the 20th training session. The addition of more training sessions seems to provide smaller BP reductions, but without statistical significance. Our results support the recommendation of the use of resistance training with benefits close to those of aerobic training in reducing the BP.
